# Permanent Electrochemical Doping of Quantum Dot Films through Photopolymerization of Electrolyte Ions

**DOI:** 10.1021/acs.chemmater.2c00199

**Published:** 2022-04-25

**Authors:** Hamit Eren, Roland Jan-Reiner Bednarz, Maryam Alimoradi Jazi, Laura Donk, Solrun Gudjonsdottir, Peggy Bohländer, Rienk Eelkema, Arjan J. Houtepen

**Affiliations:** Department of Chemical Engineering, Delft University of Technology, Van der Maasweg 9, 2629 HZ Delft, The Netherlands

## Abstract

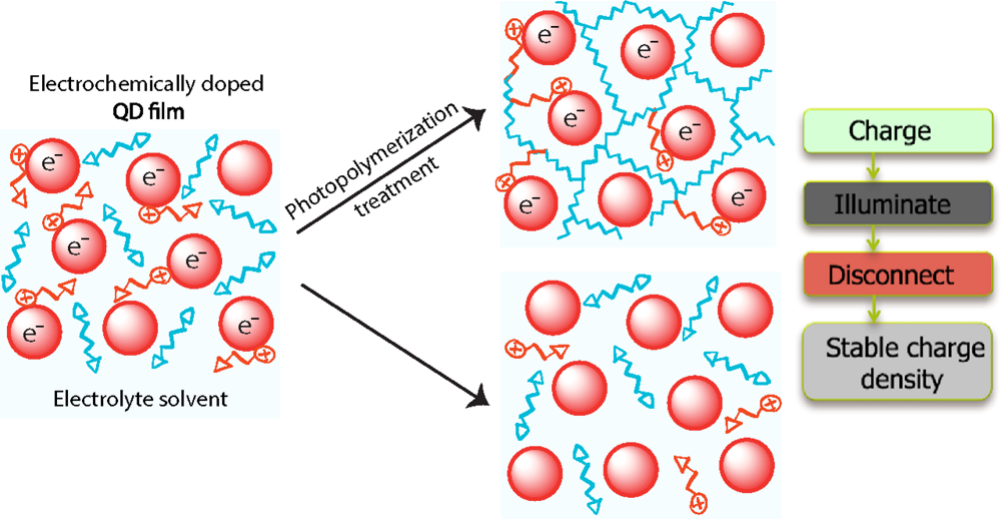

Quantum dots (QDs) are considered for devices like light-emitting diodes (LEDs) and photodetectors as a result of their tunable optoelectronic properties. To utilize the full potential of QDs for optoelectronic applications, control over the charge carrier density is vital. However, controlled electronic doping of these materials has remained a long-standing challenge, thus slowing their integration into optoelectronic devices. Electrochemical doping offers a way to precisely and controllably tune the charge carrier concentration as a function of applied potential and thus the doping levels in QDs. However, the injected charges are typically not stable after disconnecting the external voltage source because of electrochemical side reactions with impurities or with the surfaces of the QDs. Here, we use photopolymerization to covalently bind polymerizable electrolyte ions to polymerizable solvent molecules after electrochemical charge injection. We discuss the importance of using polymerizable dopant ions as compared to nonpolymerizable conventional electrolyte ions such as LiClO_4_ when used in electrochemical doping. The results show that the stability of charge carriers in QD films can be enhanced by many orders of magnitude, from minutes to several weeks, after photochemical ion fixation. We anticipate that this novel way of stable doping of QDs will pave the way for new opportunities and potential uses in future QD electronic devices.

## Introduction

Electronic doping, the deliberate introduction of impurity atoms to tune the electronic properties of bulk semiconductors, has played a central role in modern semiconductor technologies.^1−3^ The development of semiconductor nanomaterials offers new and improved applications. Colloidal semiconductor nanocrystals, also known as quantum dots (QDs), are one such semiconductor nanomaterial that can be implemented as a building block in many device applications.^4,5^ QDs have attracted considerable attention over the past decades as a result of their tunable optoelectronic properties and their facile and cheap solution-based synthesis, which makes them interesting for a wide range of applications including solar cells,^6,7^ light-emitting diodes (LEDs),^8,9^ photodetectors,^10,11^ lasers^12,13^ and thermoelectrics.^14,15^

To utilize the full potential of QDs in such optoelectronic applications, control over the charge carrier density is vital.^16−19^ Despite the maturity of electronic doping in bulk semiconductors, it has remained a long-standing challenge to reliably incorporate and manipulate electronic impurities into QDs, thus slowing their integration into optoelectronic devices.^20−22^ Some progress has been made in synthesizing n-type CdSe QDs with tin and indium precursors as impurity ions^23,24^ and p-type InP QDs with Cu impurities.^25^ Mocatta et al. have demonstrated doped InAs QDs with Cu and Ag impurities via diffusion of the metal ions into the nanocrystals resulting in cation exchange.^26^ Impurity doping of CdSe and PbSe QDs via cation exchange with Ag ions as a p-type dopant has also been reported by Norris and co-workers,^27,28^ and n-doping of CdSe QD thin films via thermal annealing of indium contacts deposited onto QDs has been demonstrated.^29^

In spite of these great efforts, the concept of impurity doping in QDs has proven challenging because of the nanometer size of QDs, which leads to new difficulties not experienced in bulk materials. Introducing even a single impurity atom into a typical 5 nm diameter QD, which consists of a few thousand atoms, yields a doping concentration of 10^19^ cm^–3^, which is already within the heavily doped limit in a bulk semiconductor; adding such a high concentration of substitutional or interstitial impurity atoms into QDs leads to significant distortions in the crystal structure.^26^ Furthermore, it has been suggested that impurity ions are easily expelled out of nanocrystals in a process termed “self-purification”,^17^ although it remains to be proven if this really plays a role below the impurity solubility limit of the bulk materials.

Doping QDs has also been demonstrated by means of chemical doping^18,30,31^ through the use of electron- or hole-donating molecules in the vicinity of the QD surface and photodoping;^32,33^ in these cases, the donor/acceptor atoms remain outside the QD crystal lattice, preventing the problems mentioned above for impurity doping. Although these external doping strategies are attractive, there remains a lack of control over the charge density and stability of the doped QDs.

**Figure 1:**
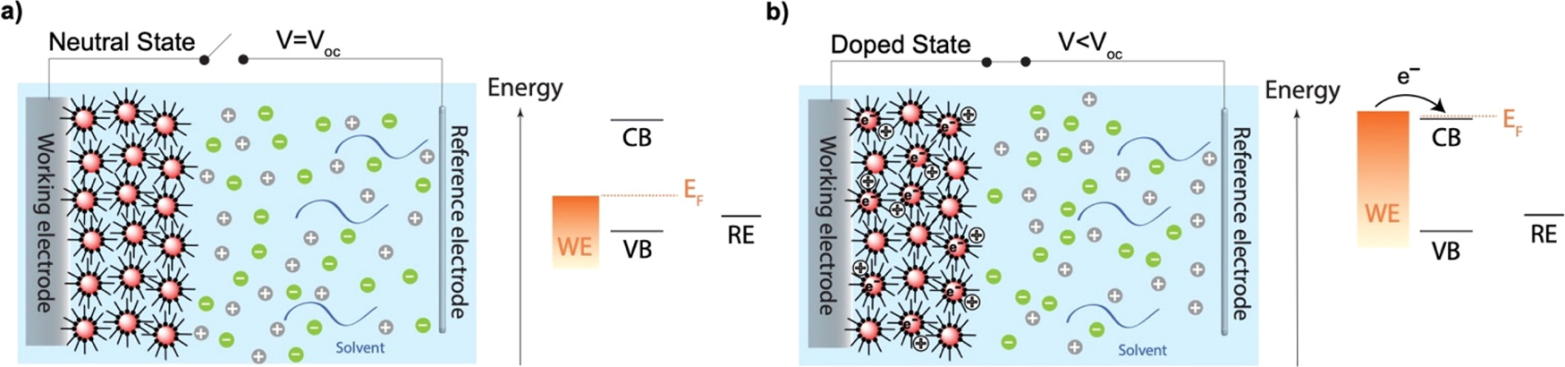
Schematic of electrochemical charge injection into QD film. (a) Situation where the Fermi level is inside the band gap of the QD when there is no applied potential to the WE, (b) situation where the Fermi level in this case is above the conduction band of the QD as a function of applied negative potential with respect to RE. To neutralize the injected electrons electrostatically, electrolyte cations diffuse into the voids of QD film.

Arguably, the most versatile method for doping QDs is to inject extra carriers by using electrochemistry.^34−37^ Electrochemical doping is an effective method that does not interfere with QD surface chemistry, nor lead to a disruption of the crystal lattice, nor introduce defect states in the band gap, as is the case for impurity doping. It allows us to adjust the charge carrier concentration precisely and controllably as a function of applied potential. Electrons or holes are injected into the QDs by externally changing the Fermi level of the sample through a potentiostat. As a result of this charge injection, electrolyte ions of opposite charge are drawn into the voids of QD film, which act as external dopants and prevent the macroscopic charging of the film, as depicted schematically in Figure 1. Efficient charge compensation by diffusing ions is allowed due to the porous nature of QD films, resulting in uniform doping of the full volume of the QD film. This contrasts with nonporous films where charge compensation is only maintained in a planar manner, causing a change of charge carrier density only near the surface in the space charge region that forms there.

While electrochemical doping offers a high level of control over the charge density, the stability of the injected charges is usually limited. When the electrochemical cell is disconnected from the potentiostat, the injected charges leave the QD film spontaneously in a matter of seconds to minutes.^38^ The disappearance of the injected charges could stem from the electrochemical side reactions with solvent impurities present in the cell environment^39,40^ or intrinsic side reactions taking place on the surface of the QDs.^41−44^ Additionally, the unbound ions in the void between the QDs will quickly migrate in an electric field, causing unwanted changes to the charge density in operating devices.^45^

Despite the versatility of electrochemical doping, little attention has been paid to the instability of injected charges. An important exception is in the field of light-emitting electrochemical cells^46−50^ (LECs), which possess the closest similarity to the electrochemically doped QD films discussed here. Several strategies have been researched to improve the doping stability in LECs. Gao et al. showed that freezing the electrolyte solvent at 100 K stabilizes the doping density in polymer LECs.^51,52^ We have recently extended this approach by using electrolyte solvents that are solid at room temperature; QD films and conducting polymers can be charged at elevated temperature and subsequently cooled down. We showed that this results in electrochemically doped QD films and conducting polymers that have a stable doping density at room temperature.^38,53^ While this shows that immobilizing the electrolyte ions stabilizes electrochemically doped systems, this stability is limited at (slightly) elevated temperatures.

A more practical way of stabilizing electrochemically injected charges is by chemically fixating the electrolyte ions after doping. For LECs, this has been investigated by using polymerizable electrolyte ions and/or molecules.^54−60^ Polymerization is induced either electrochemically or optically after charge injection. The polymerization of the solvent and electrolyte ions immobilizes the ions and probably also prevents diffusion of redox active impurities, resulting in strongly improved stability of the injected charge density. While these approaches have been investigated in the context of LECs, they have not been studied for the stabilization of electrochemically doped QD films.

In this work, we demonstrate the realization of a fixed and stable doping density in electrochemically doped ZnO and PbS QD films at room temperature via photopolymerization. By employing a dedicated polymerizable ion and electrolyte solvent system, we aim to immobilize the dopant ions photochemically after electrochemical charge injection into the QDs, which fixes the electrostatic potential caused by these ions, thus fixing the Fermi level of the system at exactly the potential determined. We monitor the change in stability for doped films by measuring the electrochemical potential and electrical conductivity over time before and after the photopolymerization treatment. We show that with this approach, the stability of charge carriers can be enhanced by many orders of magnitude, from minutes to weeks at room temperature. By performing cyclic voltammetry (CV), we demonstrate that the ionic mobility of the dopants after photochemical fixation can be lowered by many orders of magnitude. As a comparison study, we report the results from nonpolymerizable conventional electrolyte ions used in electrochemistry, such as LiClO_4_, to emphasize the greater effect of polymerizable dopant ions in both charge stability and ion immobilization. We anticipate that this novel way of stable doping of QDs could pave the way for new opportunities and potential uses in future electronic devices.

## Experimental Section

### Materials

Zinc acetate dihydrate (Zn(CH_3_COO)_2_·2H_2_O ACS reagent, ≥99.8%), potassium hydroxide (KOH, pellets EMPLURA), methanol (anhydrous ≥99.8%), ethanol (anhydrous ≥99.9%), acetonitrile (ACN, anhydrous ≥99.99%), hexane (anhydrous ≥99.8%), formamide (FA, ≥99%), lithium perchlorate (LiClO_4_, 99.99%), cadmium oxide (CdO, 99.999%), oleic acid (OA, 90%), 1-octadecene (ODE, 90%), sulfur powder (S, 99.99%), oleylamine (OLA, 70%), lead chloride (PbCl_2_, 99.99%), tetrabutylammonium iodide (TBAI, ≥99%), [2-(Acryloyloxy)ethyl]trimethylammonium chloride (ATMA-Cl, 80 wt % in H_2_O, contains 600 ppm monomethyl ether hydroquinone as inhibitor), tetra(ethylene glycol) diacrylate (TEGA, technical grade, contains 150–200 ppm MEHQ and 100–150 ppm HQ as inhibitors), poly(ethylene glycol) dimethacrylate (PEGMA-550, contains 80–120 ppm MEHQ and 270–330 ppm BHT as inhibitors), di(ethylene glycol) dimethacrylate (DEGMA, 95%, contains 300 ppm monomethyl ether hydroquinone as inhibitor), and 4,4′-bis(diethylamino)benzophenone (photoinitiator, ≥99%) were all purchased from Sigma Aldrich. ACN was dried in an Innovative Technology PureSolv Micro column before use. ATMA-Cl salt was further treated to decrease the water content by carefully heating the salt solution to 85 °C for 30 min until white salt crystals are observed. Afterward, it was attached to a vacuum line overnight at room temperature to obtain dry powder of ATMA-Cl salt, which was then transferred to a nitrogen-filled glovebox for storage. ^1^H-NMR analysis of dried ATMA-Cl salt confirmed that even after the treatment, it still retains its chemical structure without being polymerized. (See the Supporting Information, Figure S1). NMP, FA, and DEGMA were vacuum-degassed for 3 h under vigorous stirring before use and were stored in a nitrogen-filled glovebox. All other chemicals were used as received.

### Synthesis and Characterization of ZnO and PbS QDs

The synthesis of ZnO QDs was carried out under air by slight modification of two known procedures in the literature.^61,62^ Zinc acetate dihydrate (3.43 mmol) was added to 50 mL of ethanol in an Erlenmeyer flask equipped with a magnetic stir bar and heated to 60 °C. In a separate vial, potassium hydroxide (KOH) pellets (6.25 mmol) and 5 mL of methanol were combined and sonicated for 3 min at room temperature. When both reagents were dissolved completely, under constant stirring, KOH solution was added dropwise to zinc acetate solution over 10 min. The solution was then allowed to stir for one additional minute before removing the heat source. The ZnO QDs were purified by adding hexane until the solution became turbid. The flocculates were isolated by centrifugation at 2000 rpm for 1 min, and the colorless supernatant decanted.

The ZnO QDs were then redispersed in ethanol and filtered through a syringe filter (0.2 μm). The dispersion was stored at −20 °C to avoid further growth of nanocrystals. An image of a pale blue-green emission from ZnO QDs is shown in the Supporting Information, Figure S2a. Transmission electron microscopy (TEM) characterization further confirmed the successful synthesis of ZnO QDs with an average diameter size of 3.5 ± 0.2 nm, as shown in Supporting Information, Figure S2b.

PbS QDs were synthesized via a controlled cation exchange reaction from CdS QDs, where the Cd^2+^ cation is exchanged for the Pb^2+^ cation, following the procedure of Zhang et al.^63^ The synthesis was started with the preparation of CdS QDs by heating a mixture of 1 mmol (0.128 g) CdO, 3 mmol (0.942 g) OA, and 15 g of ODE for 20 min at 260 °C, then the temperature was set to 250 °C. The S precursor was made by dissolving S powder in ODE (0.5 M) at 130 °C. The S precursor (1 mL) was injected into the Cd precursor at 250 °C, and the solution was maintained at 240 °C. About 13 min later, additional S precursor was added to the solution dropwise until the desired size was achieved. The CdS QDs were washed twice with hexane and ethanol and centrifuged at 7500 rpm for 5 min. The CdS QDs were dispersed in ODE. For PbS QDs, OLA (5 mL) and PbCl_2_ (1.5 mmol) were heated at 140 °C for 30 min until a white and turbid solution was formed. Then, the solution is heated to 190 °C, and 1 mL of the CdS QDs was injected swiftly. The reaction was quenched with a water bath 20 s later, and 5 mL of hexane and 4 mL of OA were added and at 70 and 40 °C, respectively. The PbS QDs were washed 3 times using hexane and ethanol and centrifuged at 7500 rpm for 5 min. The resulting PbS QDs were dispersed in hexane. The absorption spectrum of PbS QDs is shown in the Supporting Information, Figure S3.

### Preparation of ZnO and PbS QD Films

All films were deposited on two different substrates: fluorine-doped tin oxide (FTO) and home-built interdigitated gold electrodes (IDE), both served as working electrodes (WEs) in our electrochemical cell experiments. The IDE was a glass substrate coated with three individual gold WEs with an interdigitate approach that have source-drain gaps of different sensitivities. An image of the IDE is shown in the Supporting Information, Figure S4. The ZnO QDs films were prepared by drop-casting ZnO dispersion on top of the substrate followed by annealing treatment at 60 °C for one hour in air. The PbS QDs films were prepared by dip-coating. Initially, the substrate is dipped into the QD solution, followed by dipping into the ligand solution, which in this case is TBAI (11 mg/mL) in methanol for around 30 s. After removal from the TBAI solution, the QD-coated substrate was rinsed in neat methanol for about 10 s. This process was repeated several times to build up PbS QD layers on the substrate. A Dektak profilometer was used to determine the film thicknesses, which were approximately 4 μm and 90 nm for ZnO and PbS QD films, respectively.

### (Spectro)Electrochemical Measurements

All (spectro)electrochemical measurements were performed in a nitrogen-filled glove box to ensure oxygen- and water-free conditions (≤0.1 ppm O_2_ and ≤0.5 ppm H_2_O) unless stated otherwise. An Autolab PGSTAT128N potentiostat including an additional dual-mode bi-potentiostat BA module was used to control the potential difference between the WE and the reference electrode (RE) by adjusting the current at the counter electrode (CE). The QD film was immersed in an electrochemical cell containing an electrolyte solution together with an Ag wire as the pseudoreference electrode (PRE) and Pt wire as the CE. CVs were recorded by starting near the open-circuit potential (*V*oc), with a scan rate of 50 mV/s in the negative direction until the electron injection into the conduction band of the QD film takes place followed by electrolyte ion diffusion into the voids of porous QD film for electrostatic charge compensation. As a function of the applied potential, changes in the absorption of the QD film were recorded concurrently with a fiber-based UV–Vis spectrometer, Ocean Optics USB2000. The spectroelectrochemical measurements of QD films were performed only on FTO substrates.

### Fermi-Level Stability Measurements

The stability of electrochemically injected charges was measured by performing the so-called potential vs time measurements, also known as Fermi-level stability measurements. This involves measuring the potential of the working electrode vs the reference electrode after removing the electrical connection between the WE and the CE. Any change in the potential of the system after doping will result in a change in the Fermi level of the system or vice versa. During photopolymerization of electrolyte solution, a very high electrolyte resistance between the WE and the RE is built up as the ionic conductivity drops, resulting in significant noise in the measured potential. Therefore, smoothing (Savitzky–Golay) was applied to the raw data in the Fermi-level stability measurements. The raw data set for both ZnO and PbS QD films can be seen in the Supporting Information, Figure S5.

### Conductivity Measurements

A second method to measure the stability of injected charges is to monitor the change in the conductivity of the doped QD film after removing the cell connection from the potentiostat. If injected electrons leave the conduction band of the QD film, the conductivity of the film is expected to drop as it is directly proportional to the charge density in the film. Samples of QD film deposited on IDE substrates with source-drain geometry (WE1 and WE2) were used, which enables the measurement of the electronic conductivity laterally through the film by using a Keithley 2400 SourceMeter. The width of the source-drain gap was 50 μm, and the length of the gap was 74 cm for QD films. The current was recorded over a constant 10 mV of potential difference applied through Keithley between the source and drain. The slope of the current vs potential gives the conductance, G, of the QD film. From the conductance, one can calculate the source-drain electronic conductivity σ according to the following relation:
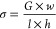
where *w* is the source-drain gap width, *l* is the gap length, and *h* is the height of the QD film. For measurements of the long-term stability of the conductivity (Figure 4e, f), a constant 10 mV source-drain bias (*V*_sd_) was applied, and the current was measured. The conductivity was obtained as *G* = *I*/*V*_sd_, assuming that background currents are negligible compared to the source-drain current.

### Photopolymerization Experiments

All photopolymerization experiments were carried out in a nitrogen-filled glovebox. A UV–LED light source (600 mW/cm^2^) with an emission wavelength of 395 nm was used to initiate the free radical chain polymerization reaction after electrochemical doping. Polymerizable electrolyte solution (0.1 M) was prepared by dissolving ATMA-Cl salt in 10 mL of FA:DEGMA (2:3 v/v) solvent mixture. FA was used to dissolve ATMA-Cl, which is an ammonium salt with a functional acrylate group at one end, and DEGMA was employed as a cross-linking agent in the photopolymerization reaction, which has bifunctional methacrylate groups on each side. The chemical structures of both monomers can be seen in Figure 3a. For experiments with nonpolymerizable electrolyte ions, solutions of 0.1 M were separately prepared by dissolving LiClO_4_ salt in 10 mL of ACN and in 10 mL of FA:DEGMA (2:3 v/v) solvent mixtures. A photoinitiator molecule (∼1 mg) was added in all experiments. A three-electrode electrochemical cell is immersed in electrolyte solution, and the electrochemical potential of the WE was set and kept exactly at −0.9 and – 0.75 V vs Ag PRE for ZnO and PbS QD films, respectively, during the entire photopolymerization experiment. This is to assure that the QD film is in the doped state while the photochemical fixation of the electrolyte solution is in progress, which then fixes the Fermi level of the system at exactly the potential specified. After 90 min of UV-light irradiation time, the electrochemical cell was disconnected from the potentiostat so that no further electron injection or extraction could occur through the external circuit. The charge stability measurements were then performed as mentioned above in the conductivity and Fermi-level stability measurement sections.

## Results and Discussion

**Figure 2:**
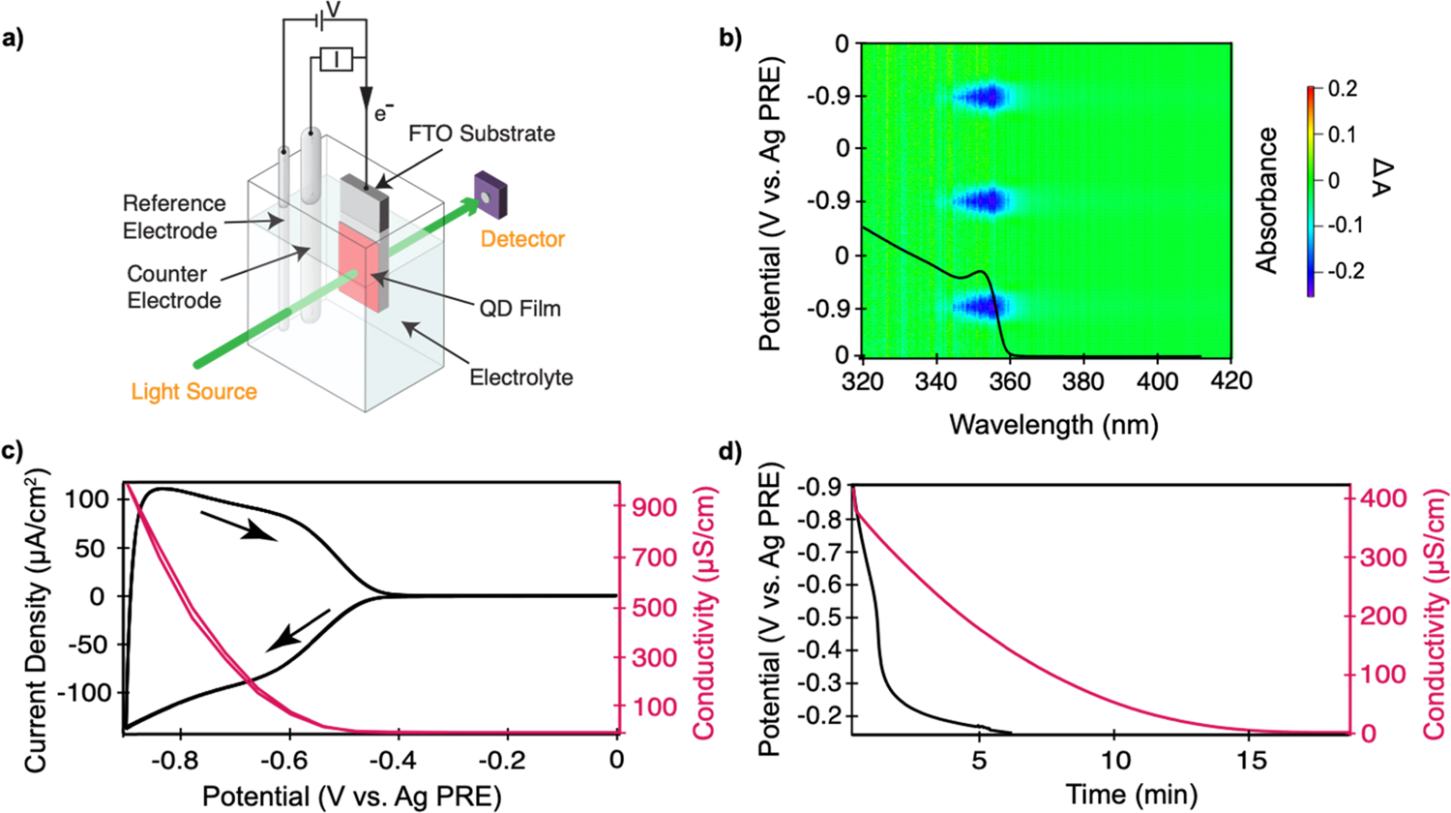
(a) Schematic of the spectroelectrochemistry cell set up with three-electrode configuration for in-situ optical absorbance measurement, (b) 2D color map showing the differential absorbance, bleach (Δ*A*) from ZnO QD film during electrochemical charging and discharging (with a scan rate of 50 mV/s), with an absorption spectrum (black line) of the film on top of it, (c) the CV of the ZnO QD film scanned starting around 0 to −0.9 V vs Ag PRE with a scan rate of 50 mV/s (black line) and the conductivity of the same film as a function of the applied potential (red line), (d) both conductivity (red line) and Fermi-level stability (black line) measurements over time after disconnecting the cell from the potentiostat. All measurements are performed in an electrolyte solution containing 0.1 M LiClO_4_ in ACN.

Electrochemical charge injection into the QD films was monitored by in-situ absorption spectroscopy in a three-electrode electrochemical cell set-up, as shown schematically in Figure 2a. As a function of applied potential, changes in the absorption of the QD film were studied. The 2D color map in Figure 2b demonstrates the differential absorbance, bleach (Δ*A*) from ZnO QD film in electrolyte solution of 0.1 M LiClO_4_ in ACN during three cycles of charging/discharging, with an absorption spectrum of ZnO QDs on top of it (black line). The blue color on the 2D map indicates the bleach of the interband optical transitions as a result of electron injection into the conduction band of the QD film. The reproducibility absorption changes upon charging/discharging reflects the high stability of the ZnO QD film as well as the easiness and controllability in tuning the Fermi level of the system with this method.

Figure 2c shows CVs of the ZnO QD film in the same electrolyte solution where the potential was scanned three times from 0 to −0.9 V vs Ag PRE with a scan rate of 50 mV/s. At more negative potentials (starting from nearly −0.45 V), the current density increases, corresponding to electron injection into the QD film. Reversing the scan direction results in an extraction of electrons from QD film. The symmetry in the CV scans indicates the absence of a significant diffusion overpotential: the charge compensating Li^+^ ions move through the voids of QD film with very little resistance during electrochemical charging and discharging. Furthermore, the high reversibility in the CV plots indicates that the large majority of the injected electrons can be extracted when the applied potential scanned back to *V*_oc_. This is not trivial because in experiments that investigate doping of QDs, it is often found that more electrons are injected than that are present in the conduction band.^32,64^

The ZnO QD films have very symmetric CVs with charge extraction ratios of typically ∼90%; see Figure 2c. These can be improved to ∼99% by very carefully drying the electrolyte, as we showed in our previous study.^41^ However, most other QD materials show much less symmetric charging and discharging curves.^38^ This is true, for instance, for CdSe QDs but also for the PbS QDs shown in the current study.

The electrical conductivity of the ZnO QD film was measured in a source-drain configuration (see Methods) as a function of applied potential and is shown in the same figure with the red line. Like the CV and the absorption bleach, the conductivity is highly reversible. The onset potential of all three measurements correlates (a Δ*A* vs voltage graph can be seen in Supporting Information Figure S6). All these effects clearly demonstrate that electron injection into the conduction band levels and dopant ion diffusion into the voids of ZnO QD film take place successfully and starts around −0.5 V vs Ag PRE.

To measure how stable the injected electrons are, we performed both Fermi-level stability and conductivity measurements vs time after breaking the connection between the WE and CE. The results are shown in Figure 2d. In the Fermi-level stability measurement, a rapid potential drop from −0.9 V to *V*oc is observed within roughly 5 min upon disconnecting the cell. Likewise, the conductivity measurement shows a similar trend with a fast decay in approximately 20 min. Hence, in spite of the high control and reversibility of the electron injection shown in Figure 2b ,c, the stability measurements in Figure 2d clearly show that the injected electrons disappear from the conduction band on the timescale of 5–10 min. Furthermore, we have previously shown that the potential decay is indeed due to the loss of conduction band electrons as it correlates exactly with a loss of the band-edge bleach. This is carried out by monitoring the differential absorbance of the QD film during Fermi-level stability measurement.^38^

We note here that a drop in charge density should not occur spontaneously. After injecting electrons, these become electrostatically bound to the electrolyte cations in the voids of the QD film. While the cations can in principle diffuse back to the bulk electrolyte solution, the electrons cannot move back to the CE if the latter is physically disconnected from the WE. This situation differs from a charged light emitting electrochemical cell mentioned in the Introduction section, where electrons and holes can move from one electrode to another through the semiconducting material. Rather, the electrochemical cell configuration should be compared to a battery that discharges spontaneously.

The fact that the electrons do not remain in the CB shows that there is another process that removes them. This could be due to impurity molecules such as dissolved O_2_ or H_2_O in the electrolyte solution that might react with the injected electrons, thus lowering the charge density in QD film. O_2_ molecules could react with electrons in the conduction band of the QD material, forming oxygen radical anions (superoxide), which may further react with water or surfactants, making the process irreversible. We previously investigated the effect of O_2_ and H_2_O on the stability of charge density in QD films and found that intentionally exposing doped QD films to oxygen causes a much quicker loss of injected electrons.^41,53^ Another possible explanation for the loss of potential and conductivity could be that conduction band electrons get trapped into states inside the band gap. However, because the Fermi level lies inside the conduction band, all trap states should already be full. There is ample time for electrons to already fill such trap states during the electrochemical charging that precedes the stability measurements. Unless electron trapping requires a very high activation energy, and happens on a timescale of minutes, we do not expect this to be an important contribution.

Indeed, the CVs of ZnO QD film measured in air differ greatly from CVs taken in the nitrogen-filled glove box environment (Supporting Information, Figure S7). The electrochemical irreversibility in the CVs of ZnO QD film measured in air clearly demonstrates that injected electrons react with oxygen even during the timescale of the CV measurement (approximately a minute). Removing all trace amounts of oxygen is thus essential for reversible electrochemical measurements and enhancing the stability of the injected electrons.^43^ However, for doped QD films that are truly stable on long timescales, this becomes impractical.

Even in an ideal scenario where there are no redox active impurities at all in the system, the dynamic nature of ions could cause problems for electrochemically doped systems that are used in devices. If we take the example of a pn junction used as photodiode, this would involve the application of a reverse bias for efficient charge extraction. However, that bias would also cause the electrolyte ions to migrate and would hence strongly change the doping density.

**Figure 3:**
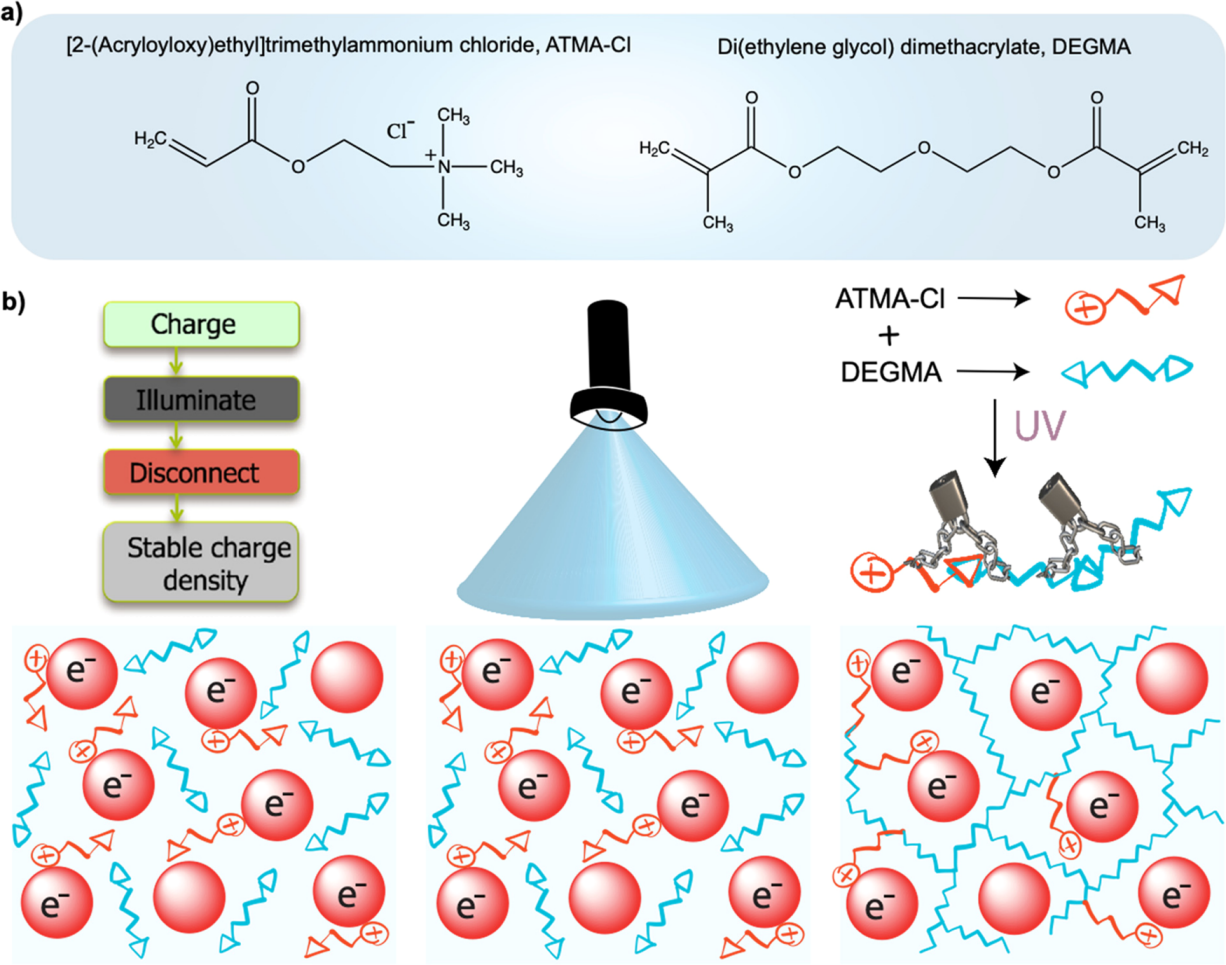
(a) Chemical structures of both compounds used for the photopolymerization experiment, (b) schematic of photopolymerization treatment with a UV-LED light source (600 mW/cm^2^) with an emission wavelength of 395 nm for chemical immobilization of the electrolyte ions after the electrochemical charge injection into the QDs.

With this in mind, we attempt to mitigate both problems (i.e., impurity diffusion and ion diffusion) by photopolymerizing the solvent and electrolyte ions in films of electrochemically doped ZnO and PbS QDs. This should bind the electrolyte ions covalently after the electrochemical charge injection into the QDs, fixing the electrostatic potential caused by these ions and consequently the Fermi level at the potential used during the polymerization, as shown schematically in Figure 3. The diffusion of impurities, which causes instability by scavenging the injected charges, will also be substantially hindered because of the formation of a dense polymer matrix, thus enhancing the stability of electrochemically injected charges inside the QD film at room temperature.

The details of the photopolymerization procedure are discussed in the Methods section. We chose to employ acrylate photopolymerization because it is simple and reliable. The formation of a densely packed polymer network is crucial for mitigating the diffusion of both electrolyte ions and impurity molecules. To this end, we tested a range of cross-linking molecules with different lengths to observe the effect on charge stability in electrochemically doped QD films, namely, di(ethylene glycol) dimethacrylate (DEGMA), tetra(ethylene glycol) diacrylate (TEGA), and poly(ethylene glycol) dimethacrylate (PEGMA-550). As the length of the cross-linking molecule increases, it is expected to result in a less-dense polymer matrix. We found the cross-linking molecule that gave the best stability was DEGMA (see the Supporting Information, Figure S12), therefore, we focus on this solvent for the remainder of this work. As polymerizable electrolyte ions, we used ATMA-Cl. Because we focus on n-doping in this work, it is sufficient if the charge compensating cation is polymerizable, although a salt that consists of both polymerizable cations and anions would be ideal, for example, in an electrochemically doped pn junction used in light-emitting electrochemical cell. To mix DEGMA and ATMA-Cl, a small amount of formamide is added. Finally, we employ 4,4′-bis(diethylamino)benzophenone as the photoinitiator.

**Figure 4:**
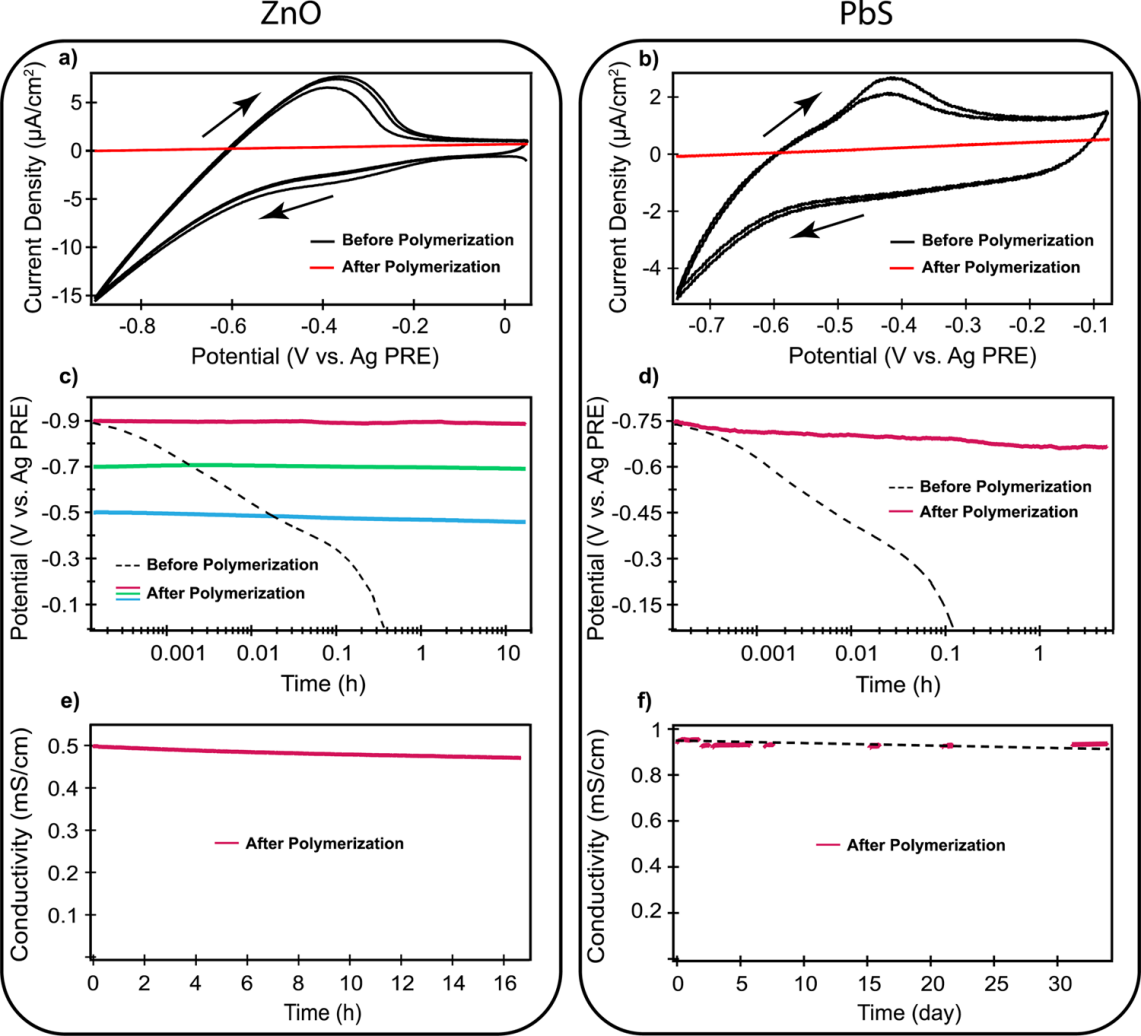
(a, b) CVs of the ZnO and PbS QD films with a scan rate of 50 mV/s before and after photopolymerization, (c, d) Fermi-level stability measurements before and after photopolymerization when disconnecting the cell from the potentiostat, (e, f) conductivity measurements of both doped films after photopolymerization upon disconnecting the cell from the potentiostat. The dashed line in f is only to guide to the eye.

ZnO or PbS QD films are charged negatively in this electrolyte solution. Figure 4a, b shows the CVs of ZnO and PbS QD films, respectively, before and after the photopolymerization treatment. The CVs before photopolymerization show clear signs of electron injection into the QD films. If we compare the charging of the ZnO QD film in this electrolyte solution (Figure 4a) with the charging in acetonitrile (Figure 2c), we notice that the former is less symmetric, and the current density is much lower. This is a result of the much higher viscosity of DEGMA than that of acetonitrile and the use of much bulkier cations (ATMA vs Li). This strongly slows down the transport of the cations through the film, which limits the current and causes a diffusion overpotential. More symmetric CVs obtained at slower scan rates support this argument. However, although charge injection is slower, it is still possible to dope the ZnO QD film *n* type using this electrolyte solution, as verified with the increase in the conductivity (see Figure 4e, f) and the appearance of a band edge absorption bleach (see Supporting Information Figure S9).

The QD films were photopolymerized while a negative potential was applied (−0.9 V for the ZnO QD film, −0.75 V for the PbS QD film). The CVs after photopolymerization are shown as the red lines in Figure 4a, b. Clearly, the polymerization has a strong effect on the CV. The current density drops dramatically, and what remains is a linear Ohmic response that is 0 at the applied potential during photopolymerization (see Supporting Information Figure S8 for a zoom in of the CVs after polymerization). We attribute this to the immobilization of the ions. Their diffusion coefficient becomes so low after polymerization that charging and discharging are no longer possible.

The results shown so far could potentially be explained by the polymerization of the DEGMA solvent alone. It is conceivable that this also reduces the diffusion coefficient of the ATMA ions. To investigate the relevance of using photopolymerizable ions instead of nonpolymerizable conventional electrolyte ions, we performed the same experiment on a film of ZnO QDs, but with 0.1 M LiClO_4_ as the electrolyte, instead of ATMA-Cl. The CVs before and after photopolymerization and Fermi-level stability measurement are shown in Figure S10 in the Supporting Information. In this case, there is only a fourfold reduction in the current density observed in the CV. This points out that the polymerization of the solvent reduces the diffusion coefficient of the Li^+^ ions, but they are still able to move through the film and cause charging and discharging of the ZnO QDs. In line with this observation, the potential decay over a 20 h (Figure S10, bottom) is much more significant than when ATMA-Cl is used as the supporting electrolyte (Figure 4c). This shows that the polymerization of the charge-compensating ATMA ions together with the DEGMA matrix has a strong positive effect on the stability of the doping density.

Figure 4c,d shows the Fermi-level stability measurements for both ZnO and PbS QD films before (dashed lines) and after (solid lines) the photopolymerization treatment. For the ZnO film before photopolymerization (dashed line in Figure 4c), the result is very similar to what was shown in Figure 2 for charging in acetonitrile. The potential drops from the charging potential of −0.9 V to *V*_oc_ in approximately 20 min. For the PbS QD film, it takes a slightly shorter time (around 7 min) for the potential to decay to *V*_oc_. After the photopolymerization treatment, the charge stability for both ZnO and PbS QD films is substantially improved, as shown by the red lines in Figure 4c, d. The Fermi level in the ZnO QD film stays constant over roughly 17 h of measurement after disconnecting the cell. For the PbS QD film, there is a minor decrease in potential, from −0.75 to −0.66 V over 5 h.

The ability to precisely adjust the charge carrier concentration in QDs as a function of applied potential will pave the way for practical implementation of QDs into optoelectronic devices, in particular, for situations where moderate doping levels are desired. To demonstrate the ability to control and fix the Fermi level at any desired potential, we used various potentials during the photopolymerization of ZnO QD films. The results are shown as red (−0.9 V during polymerization), green (−0.7 V), and blue (−0.5 V) lines in Figure 4c. The results show that the Fermi level can indeed be stabilized at any desired potential, or equivalently, at any desired charge density. As shown in the Supporting Information, Figure S11, the three potentials used correspond to electron densities of 3.6 × 10^16^ cm^–3^, 1.8 × 10^17^ cm^–3^, and 3.1 × 10^18^ cm^–3^_._

As a final test of the long-term stability of the injected electrons after the photopolymerization treatment, we measured the electronic conductivity of the doped and photopolymerized films. The results are shown in Figure 4e, f. The conductivity measurement in Figure 4e demonstrates that the charge density in the ZnO QD film after photopolymerization is stable over 17 h of measurement. During this time, there is a drop in conductivity of only 4%. The conductivity of a film of PbS QDs was measured for 33 days. The results, shown in Figure 4f, show that the drop in conductivity during this period is less than 2%, indicating that the stability of the injected electrons has increased from minutes to weeks because of the photopolymerization of the solvent and charge compensating cations. In the studies described above, we have focused on the stability of electrochemically doped QD films. In principle, the same approach could also be applied to QDs that are doped in solution, either via electrochemistry, chemical doping, or photodoping. Photopolymerization of the solvent and counter ions after doping the QD in solution could prepare a system of doped, but isolated QDs. Furthermore, the experiments above describe the stability of doped QD films inside an electrolyte solution. These results can be extended to stable doped films outside of the electrolyte solution, which may be more useful for most applications. Initial proof-of-principle experiments have shown that taking out a doped film from the electrolyte solution, followed by immediate photopolymerization, results in a similar increase in the stability of the conductivity.

## Conclusions

In summary, we showed that electrochemical doping of QD films is reversible, and controllable, but usually not stable. The injected charges leave the QD films spontaneously in minutes when the electrochemical cell is disconnected from the potentiostat. This instability is likely due to reactions with trace amounts of O_2_ in the electrolyte solution. The stability of the injected charge carrier can, however, be strongly enhanced using photopolymerization after electrochemical charge injection. By employing a dedicated polymerizable ion and an electrolyte solvent system, we demonstrated that the stability of the electron in electrochemically doped ZnO and PbS QDs is increased from minutes to weeks. The photopolymerization covalently links the electrolyte ions to the polymer matrix and fixes the electrostatic potential. Furthermore, it likely also results in the reduced diffusion of impurity ions. We showed that the ionic mobility of the dopant ions after photochemical fixation can be significantly lowered, preventing and further charging or discharging the QD films. Results with nonpolymerizable conventional electrolyte ions show only a marginal improvement of the stability with magnitude from min to several weeks after photochemical ion fixation at room temperature. An additional advantage of using photopolymerization to stabilize the injected charges is that it may provide a path toward patterning the doping density and forming junctions on demand in the QD films. We anticipate that this novel way of doping QDs will pave the way for new opportunities and potential uses in future QD electronic devices.

## Supporting Information Available

^1^H-NMR spectrum of dried ATMA-Cl salt, image of a pale blue-green emission from ZnO QDs, TEM image of synthesized ZnO QDs, absorption spectrum of PbS QDs, image of the interdigitated gold electrode, raw data set from voltage vs time experiments, differential absorbance (Δ*A*) vs voltage graph of ZnO QD film, CV of ZnO QD film scanned outside the glovebox, CVs of the ZnO and PbS QD films after photopolymerization treatment, 2D color map showing the differential absorbance, bleach (Δ*A*) from ZnO QD film during electrochemical charging and discharging, CVs of the ZnO QD film before and after photopolymerization treatment and Fermi-level stability measurement in an electrochemical cell containing electrolyte solution of LiClO_4_ in the FA:DEGMA solvent mixture, differential capacitance measurements performed on ZnO QD film, Fermi-level stability measurements of the ZnO QD film in an electrochemical cell containing 0.1 M ATMA-Cl in different lengths of cross-linking molecules, namely, DEGMA, TEGA, and PEGMA-550 all together with the FA solvent (PDF)
