# Supplementary material for: Permanent Electrochemical Doping of Quantum Dot Films through Photopolymerization of Electrolyte Ions
Source: Chem Mater. 2022 Apr 25;34(9):4019–28. doi: 10.1021/acs.chemmater.2c00199 (PMC9097154; doi:10.1021/acs.chemmater.2c00199)
Supplement: Supplementary file 1 — cm2c00199_si_001.pdf [file cm2c00199_si_001.pdf]

Supporting Information for -

Permanent Electrochemical Doping of Quantum Dot Films through Photopolymerization of Electrolyte Ions

Hamit Eren[1], Roland Jan-Reiner Bednarz[1#], Maryam Alimoradi Jazi[1], Laura Donk[1&], Solrun Gudjonsdottir[1], Peggy Bohländer[1], Rienk Eelkema[1] and Arjan J. Houtepen[1]*

[1]Department of Chemical Engineering, Delft University of Technology, Van der Maasweg 9, 2629 HZ Delft, The Netherlands

#Current address: Department of Chemistry, Johannes Gutenberg University Mainz, Duesbergweg 10–14, 55128 Mainz (Germany)

&Current address: Department of Chemical Engineering and Chemistry, Eindhoven University of Technology, P.O. Box 513, 5600 MB Eindhoven, The Netherlands

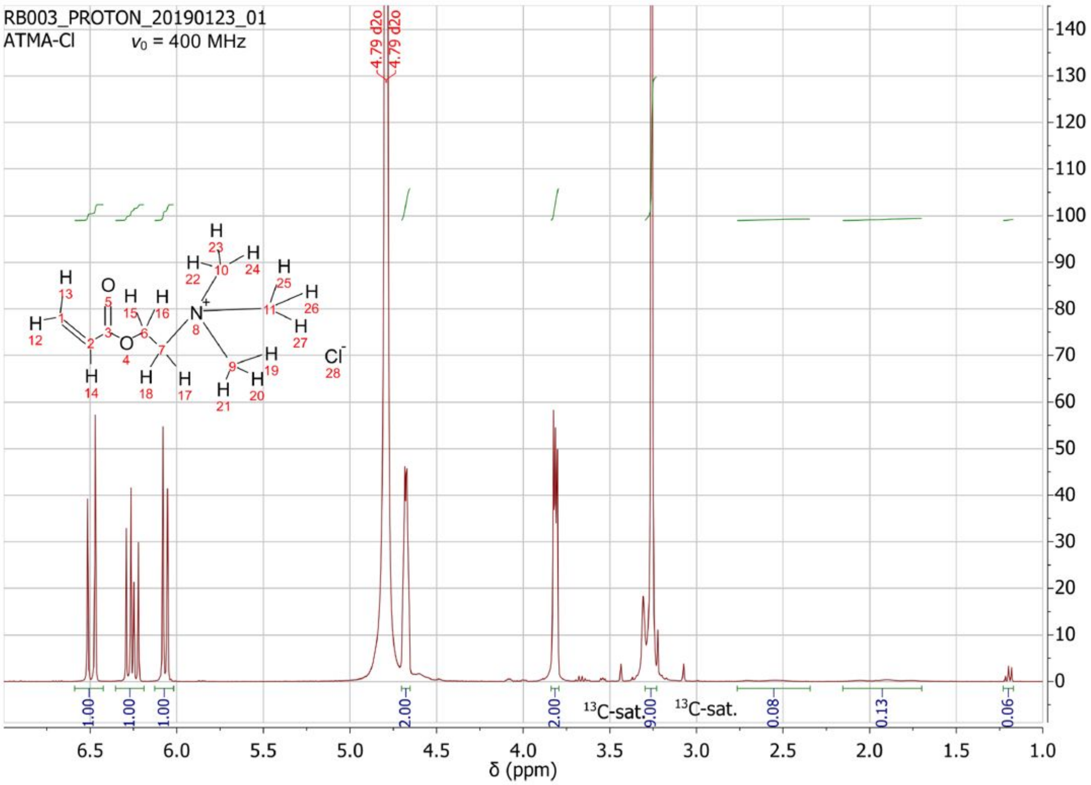

Figure S1. ^{1}H-NMR spectrum of dried ATMA-Cl salt in D_2O. In the present ^{1}H-NMR, the ATMA-Cl signals are analysed. ^{1}H-NMR (400 MHz, D_2O, 20 °C): δ= 6.49 (dd, 1H, $^3J_{trans}$(^{1}H; ^{1}H) = 17:3 Hz, ^{2}J(^{1}H; ^{1}H) = 1:0 Hz; Vinyl-CH position 1), 6.26 (dd, ^{1}H, $^3J_{trans}$ (^{1}H; ^{1}H) = 17:3 Hz, $^3J_{cis}$(^{1}H; ^{1}H) = 10.6 Hz; Vinyl-CH, position 2), 6.07 (dd, 1H, $^3J_{cis}$(^{1}H; ^{1}H) = 10.6 Hz, ^{2}J(^{1}H; ^{1}H) = 1:0 Hz; Vinyl-CH, position 1), 4.68 (m, 2H; CH_2, position 6), 3.81 (m, 2H; CH_2, position 7) and 3.26 ppm (s, 9H; 3 CH_3, positions 9 - 11).

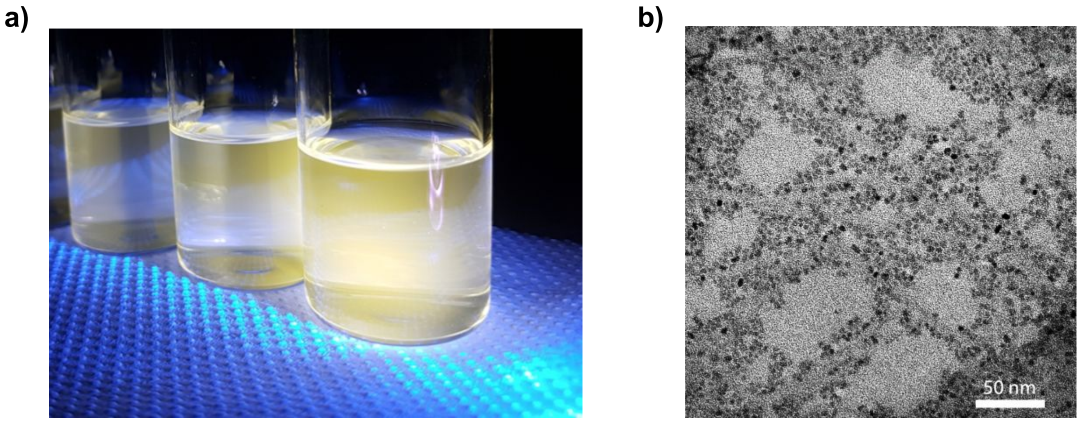

Figure S2. An image of a pale blue-green emission from ZnO QDs in ethanol (a), Transmission electron microscopy (TEM) image of synthesized ZnO QDs with an average diameter size of 3.5±0.2 nm.

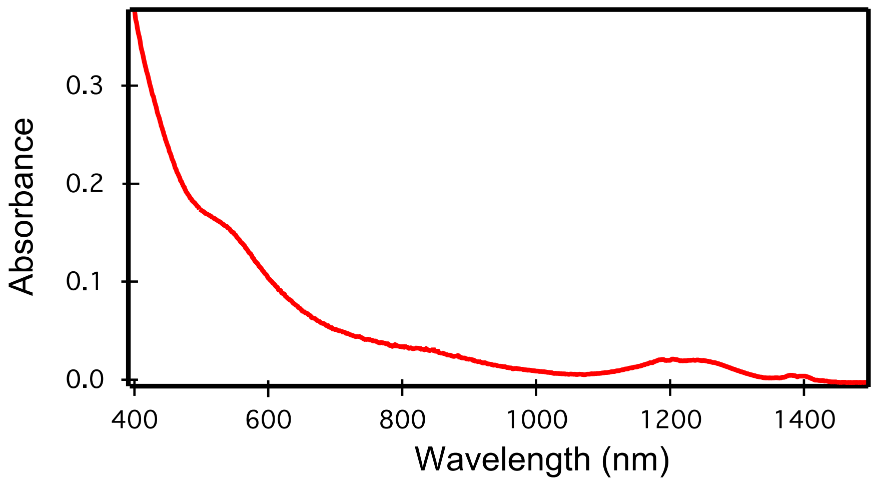

Figure S3. The absorption spectrum of PbS QDs dispersed in hexane.

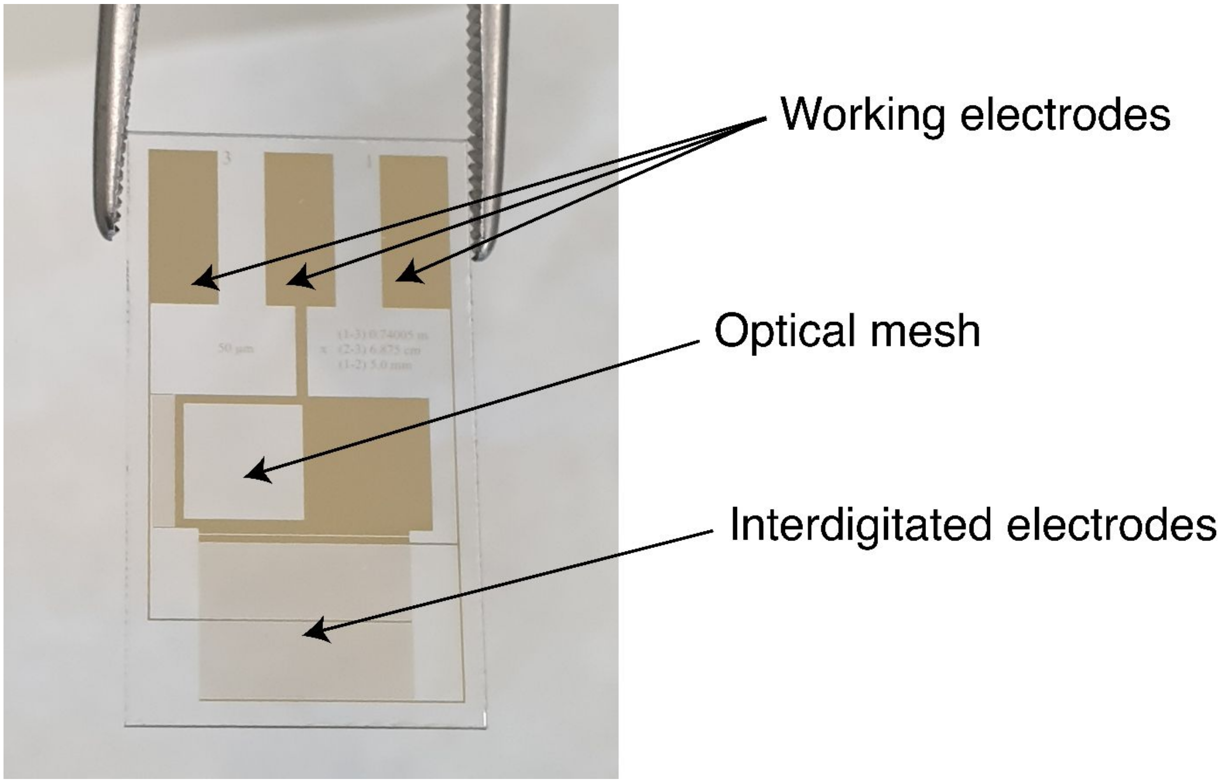

Figure S4. Home-built interdigitated gold electrode (IDE). The electrode is a glass substrate coated with three separate gold WEs which provide three source-drain gaps of different sensitivities.

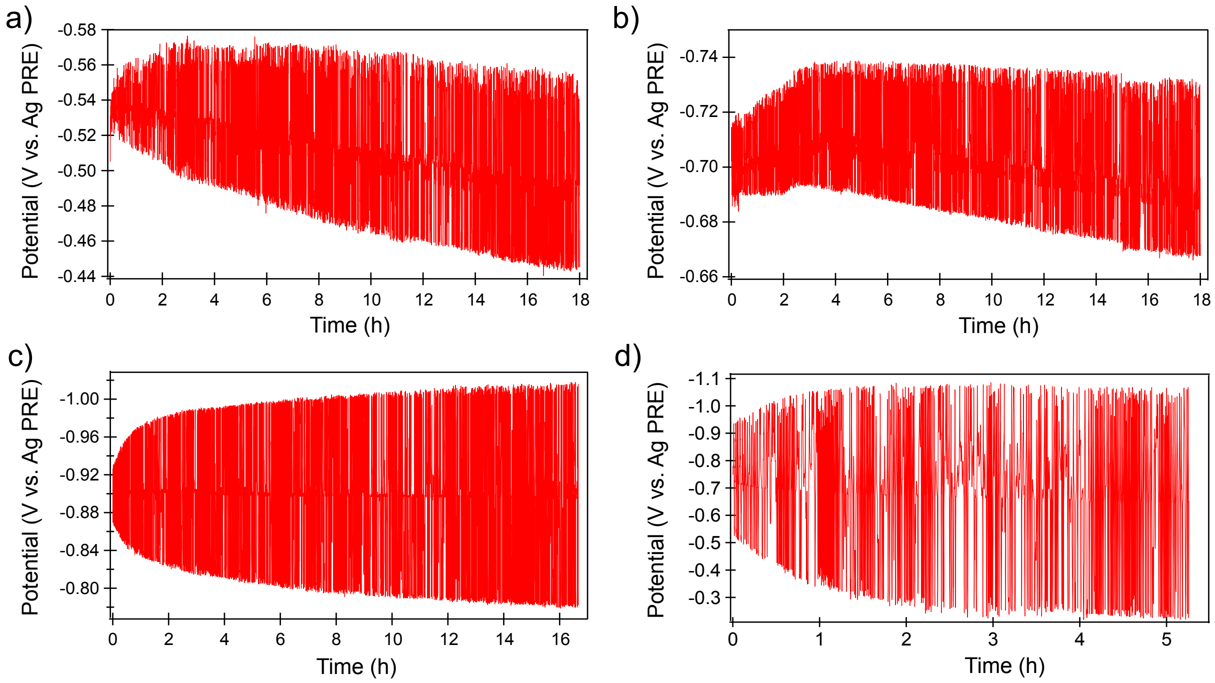

Figure S5. The raw data set obtained from voltage vs time experiments (Fermi-level stability measurements) for different potential values, namely: -0.5 V (a), -0.7 V (b), -0.9 V (c) vs Ag PRE for ZnO and -0.75 V vs Ag PRE (d) for PbS QD films.

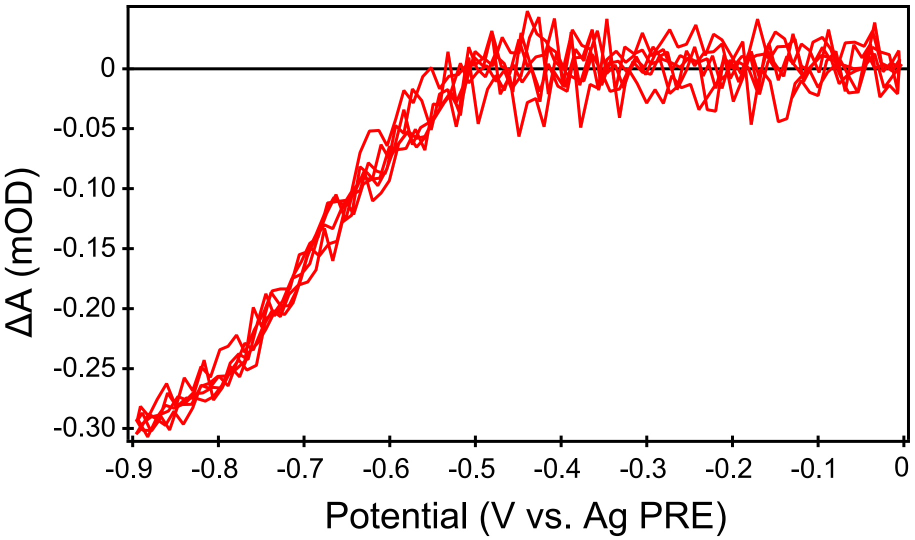

Figure S6. Differential absorbance (ΔA) vs voltage graph of the ZnO QD film in an electrolyte solution of 0.1 M $LiClO_4$ in ACN. Simultaneously monitoring the changes in absorption as a function of applied potential gives bleach features.

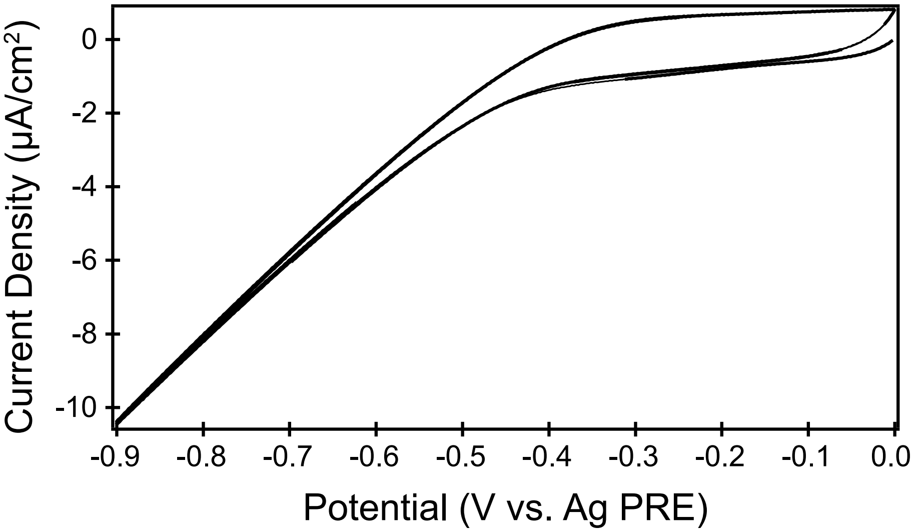

Figure S7. CV of ZnO QD film scanned outside of the glove box in an electrochemical cell containing 0.1 M $LiClO_4$ in ACN.

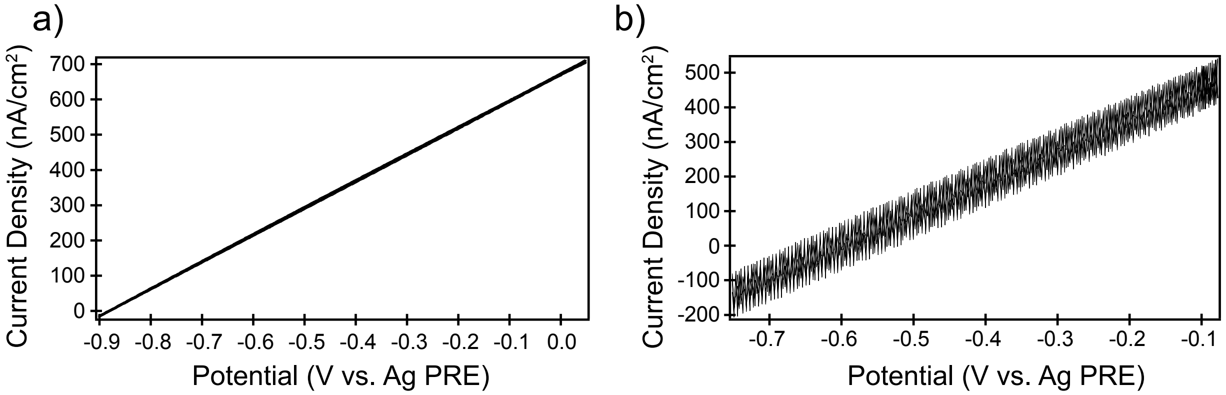

Figure S8. CVs of the ZnO (a) and PbS QD films (b) after photopolymerization treatment. As shown in the CVs, only negligible amount of current (nanoampere) is observed during the CV scans after polymerization.

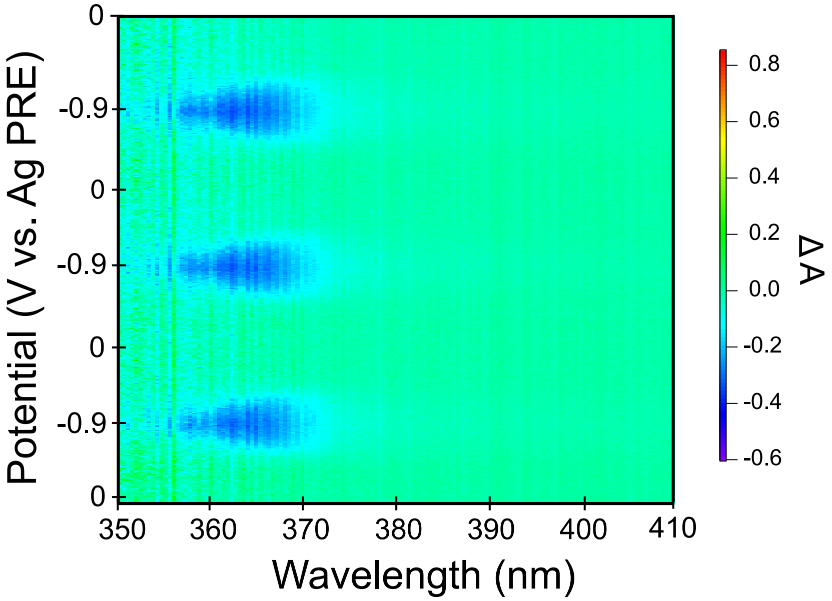

Figure S9. The 2D color map showing the differential absorbance, bleach (ΔA) from ZnO QD film during electrochemical charging and discharging in an electrochemical cell containing 0.1 M ATMA-Cl in FA.

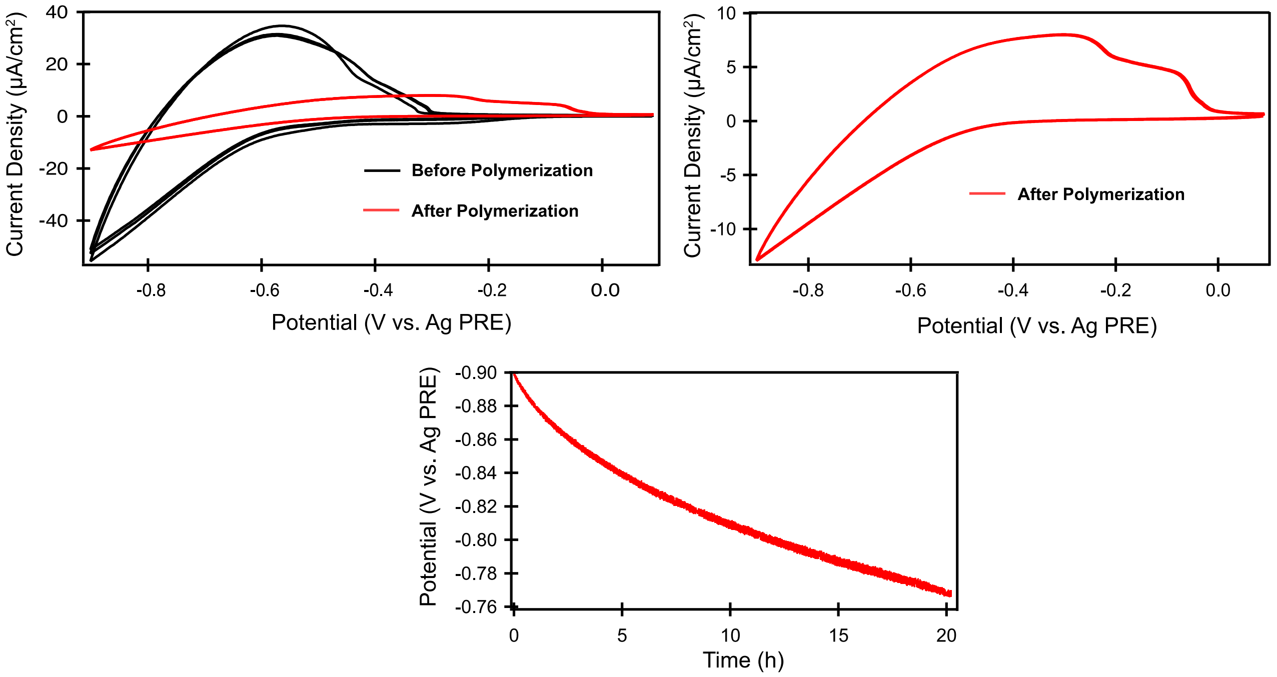

Figure S10. CVs of the ZnO QD film before (top right) and after photopolymerization treatment (top left) and Fermi level stability measurement after polymerization (bottom) in an electrochemical cell containing 0.1 M $LiClO_4$ in FA:DEGMA solvent mixture.

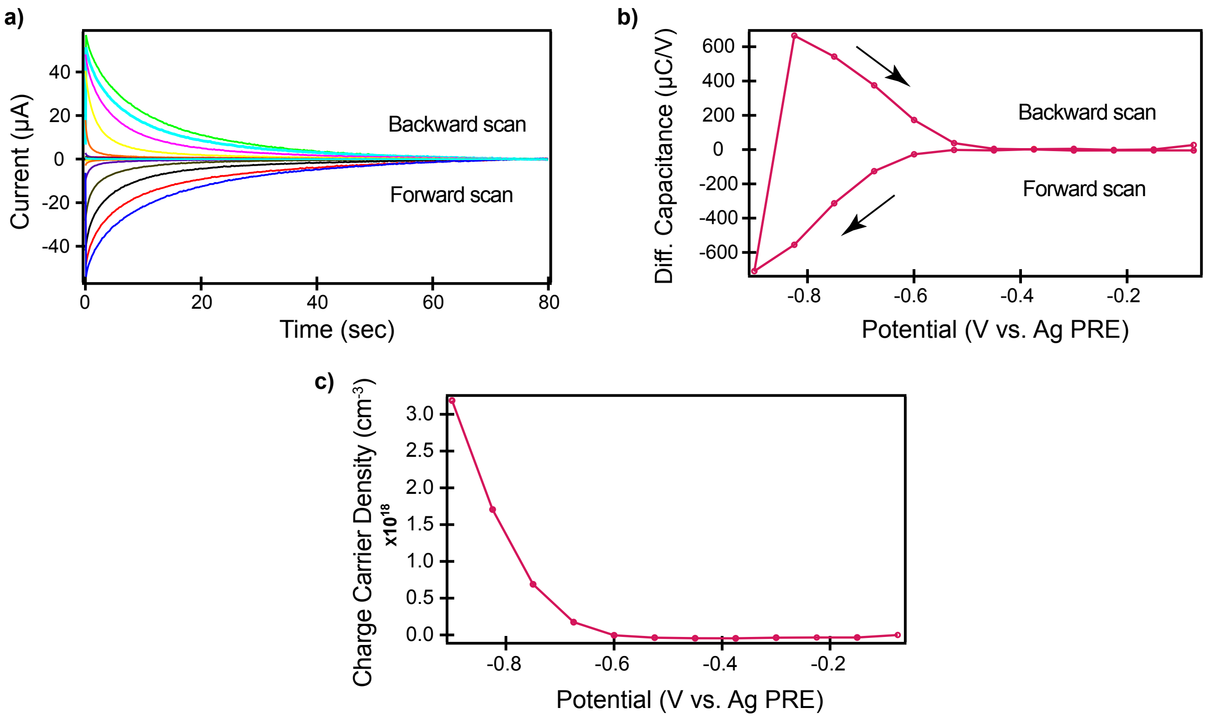

Figure S11. Differential capacitance measurements performed on ZnO QD film deposited on IDE substrate in an electrochemical cell containing 0.1 M ATMA-Cl in FA:DEGMA solvent mixture. Potential steps of 75 mV were applied and after each potential step the charging current was recorded for 80 seconds. In all cases, the initial peak currents decayed in an exponential manner to a constant current which can be assigned to background current of the electrolyte which then subtracted to obtain the electrochemical charging of the QD film (a), the integration of this charging current followed by a division of each potential step gives the differential capacitance of the ZnO QD film with a unit of coulomb per volt (b), the total injected charge was calculated by multiplying the differential capacitance with the potential applied which then divided into the film volume in order to derive the charge carrier density of the ZnO QD film as a function of applied potential (c). For example; the charge carrier density of the ZnO QD film at the potential of -0.9 V is ~10^{18} per cm^3.

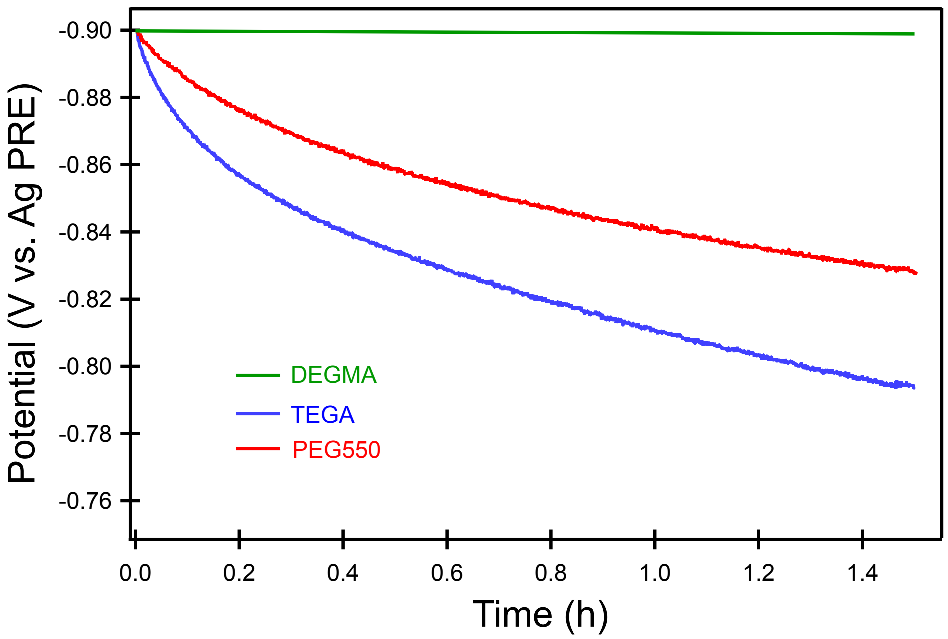

Figure S12. Fermi level stability measurements of the ZnO QD film in an electrochemical cell containing 0.1 M ATMA-Cl in different length of cross-linking molecules namely, DEGMA, TEGA and PEG550 all together with FA solvent.
